# Complete genome sequence of the Antarctic *Halorubrum lacusprofundi* type strain ACAM 34

**DOI:** 10.1186/s40793-016-0194-2

**Published:** 2016-09-10

**Authors:** Iain J. Anderson, Priya DasSarma, Susan Lucas, Alex Copeland, Alla Lapidus, Tijana Glavina Del Rio, Hope Tice, Eileen Dalin, David C. Bruce, Lynne Goodwin, Sam Pitluck, David Sims, Thomas S. Brettin, John C. Detter, Cliff S. Han, Frank Larimer, Loren Hauser, Miriam Land, Natalia Ivanova, Paul Richardson, Ricardo Cavicchioli, Shiladitya DasSarma, Carl R. Woese, Nikos C. Kyrpides

**Affiliations:** 1 DOE Joint Genome Institute, Walnut Creek, CA 94598 USA; 2 Institute of Marine and Environmental Technology, Columbus Center, University of Maryland School of Medicine, University System of Maryland, Baltimore, MD 21202 USA; 3 DOE Joint Genome Institute, Los Alamos National Laboratory, Los Alamos, NM 87545 USA; 4 Oak Ridge National Laboratory, Oak Ridge, TN 37830 USA; 5 School of Biotechnology and Biomolecular Sciences, The University of New South Wales, Sydney, NSW 2052 Australia; 6 B103 Chemical and Life Sciences Laboratory, University of Illinois at Urbana-Champaign, MC-110, 601 South Goodwin Avenue, Urbana, IL 61801 USA

**Keywords:** Archaea, Halophile, *Halorubrum*, Extremophile, Cold adaptation, Tree of life

## Abstract

*Halorubrum lacusprofundi* is an extreme halophile within the archaeal phylum *Euryarchaeota*. The type strain ACAM 34 was isolated from Deep Lake, Antarctica. *H. lacusprofundi* is of phylogenetic interest because it is distantly related to the haloarchaea that have previously been sequenced. It is also of interest because of its psychrotolerance. We report here the complete genome sequence of *H. lacusprofundi* type strain ACAM 34 and its annotation. This genome is part of a 2006 Joint Genome Institute Community Sequencing Program project to sequence genomes of diverse *Archaea*.

## Introduction

**Fig. 1:**
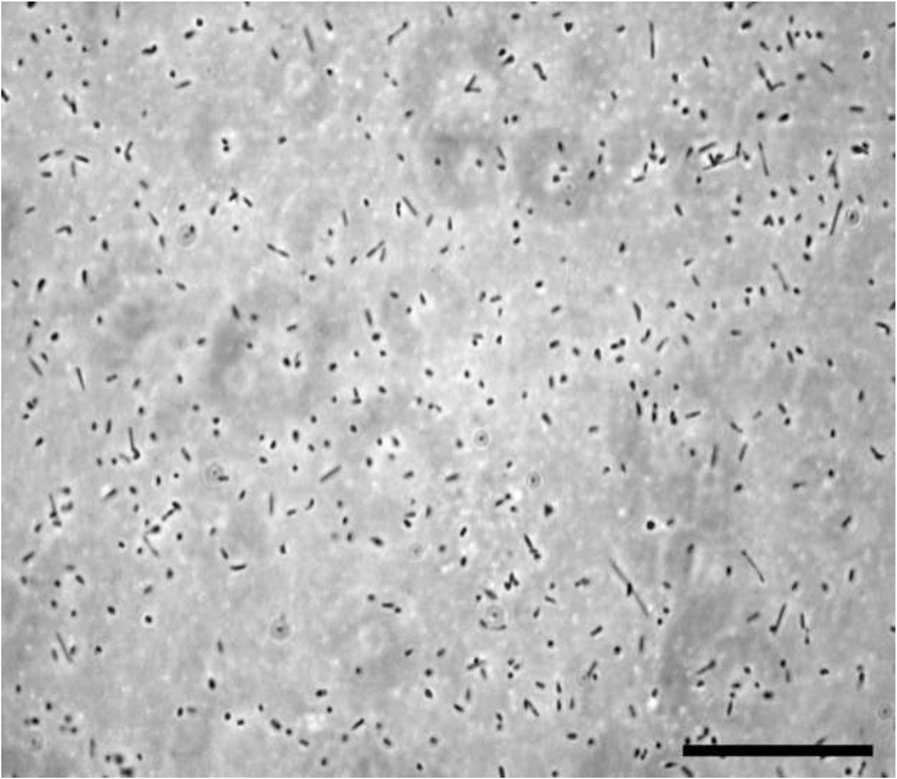
Photomicrograph of *H. lacusprofundi* type strain ACAM 34 cells. The cells were grown in Franzmann et al. [1] medium. The image was taken using a phase microscope (Nikon Labphot) with 1000× magnification. The scale bar represents 10 μm

**Fig. 2:**
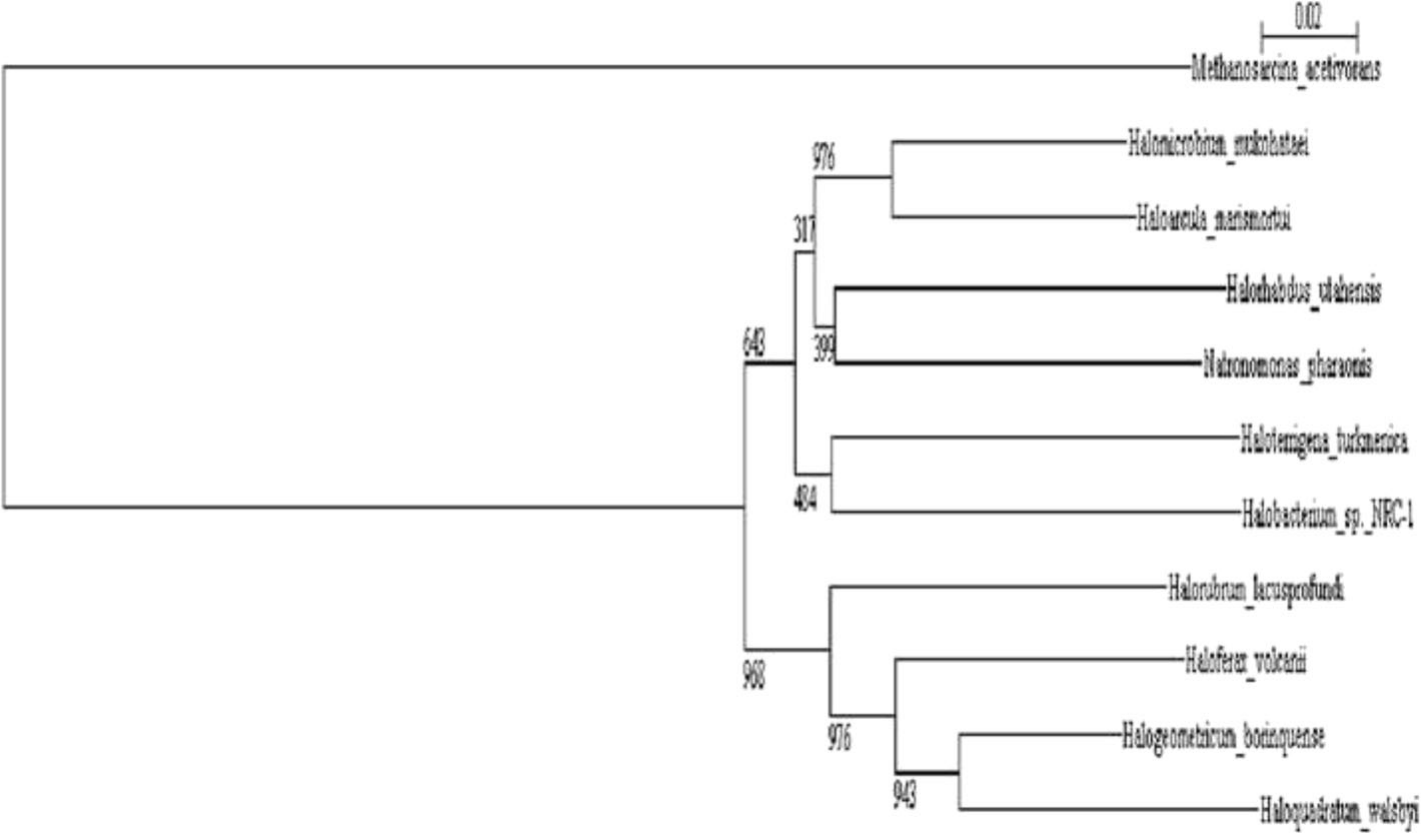
Phylogenetic tree of DNA-directed RNA polymerase subunit A’ of select haloarchaea. Sequence alignment and tree construction were carried out with Clustal W [39]. The tree was visualized with njplot [40]. Positions with gaps were excluded during tree construction. *Methanosarcina acetivorans* was used as the outgroup. The numbers indicate bootstrap values based on 1000 replicates

*Halorubrum lacusprofundi* is an extremely halophilic archaeon belonging to the class *Halobacteria* within the phylum *Euryarchaeota*. The species is represented by the type strain, ACAM 34 (= DSM 5036 = ATCC 49239 = JCM 8891), and a second strain, ACAM 32, both of which were isolated from Deep Lake, Antarctica [1]. This organism was first described as *Halobacterium lacusprofundi* but was later transferred to the genus *Halorubrum* [2]. Members of the genus *Halorubrum* have been found not only in Antarctica, but also in Africa [3], Asia [4], and North America [5], where they are usually found in saline lakes or salterns. Most members of the genus are neutrophiles, but some are haloalkaliphiles [6, 7]. *H. lacusprofundi* (Fig. 1) was proposed for sequencing as part of a 2006 Joint Genome Institute Community Sequencing Program project because of its ability to grow at low temperature and its phylogenetic distance from other halophiles with sequenced genomes (Fig. 2).

## Organism information

### Classification and features

*Halorubrum lacusprofundi*ACAM 34 was isolated from a water-sediment sample from Deep Lake, Antarctica [1]. The water-sediment sample was incubated in the light at 18 °C, and after 3 months developed a reddish color. *H. lacusprofundi* was isolated from the sample by streaking on Deep Lake vitamin agar, which was composed of Lake Deep water with 1 g/L yeast extract, 15 g/L agar, and vitamin solution. The physiological characteristics of *H. lacusprofundi* were described as follows [1]. Cells were pleomorphic. Motility was not observed, and no flagella were present. Cells grew at a temperature range of −1 °C to 40 °C with an optimal growth temperature of 36 °C [8]. Growth was observed at NaCl concentration of 1.5 M to 4.5 M with an optimum salt concentration of 3.5 M. Cells lysed in distilled water. The optimum magnesium concentration for growth was 0.1 M. No growth was observed at magnesium concentrations of 0 M or 1.0 M. Ammonium could not be used as a nitrogen source; complex media such as yeast extract or peptone was required. Growth was stimulated by addition of glucose, galactose, mannose, ribose, lactose, glycerol, succinate, lactate, formate, acetate, propionate, and ethanol. Growth was not stimulated by addition of glycine. Acid was not produced from sugars.

## Genome sequencing information

### Genome project history

*H. lacusprofundi* was selected for sequencing based upon its phylogenetic position relative to other haloarchaea and its cold tolerance (Table 1). It is part of a 2006 Joint Genome Institute Community Sequencing Program project that included six diverse archaeal genomes. Sequencing was done at the JGI Production Genomics Facility. Finishing was done at Los Alamos National Laboratory. Annotation was done at Oak Ridge National Laboratory and JGI. The complete genome sequence was finished in September, 2008 and was released to the public in GenBank in February, 2009. A summary of the project information is shown in Table 2.

**Table 1:** Classification and general features of *Halorubrum lacusprofundi* ACAM 34^T^ [31]

MIGS ID	Property	Term		Evidence code^a^
	Classification	Domain	*Archaea*	TAS [32]
		Phylum	*Euryarchaeota*	TAS [33, 34]
		Class	*Halobacteria*	TAS [35]
		Order	*Halobacteriales*	TAS [36]
		Family	*Halobacteriaceae*	TAS [37]
		Genus	*Halorubrum*	TAS [3]
		Species	*Halorubrum lacusprofundi*	TAS [1]
	Gram stain	Unknown		
	Cell shape	Pleomorphic		TAS [1]
	Motility	Non-motile		TAS [1]
	Sporulation	Nonsporulating		NAS
	Temperature range	−1–40 °C		TAS [1]
	Optimum temperature	36 °C		TAS [1]
	pH range, optimum	Unknown		
	Carbon source	Sugars, organic acids, ethanol		TAS [1]
MIGS-6	Habitat	Saline lake		TAS [1]
MIGS-6.3	Salinity	10–25 % NaCl		TAS [1]
MIGS-22	Oxygen requirement	Aerobic		TAS [1]
MIGS-15	Biotic relationship	Free-living		TAS [1]
MIGS-14	Pathogenicity	Non-pathogen		NAS
MIGS-4	Geographic location	Deep Lake, Antarctica		TAS [1]
MIGS-5	Sample collection	1988		TAS [1]
MIGS-4.1 MIGS-4.2	Latitude-Longitude	Unknown		
MIGS-4.4	Altitude	Unknown		

^a^Evidence codes–*IDA* Inferred from Direct Assay, *TAS* Traceable Author Statement (i.e., a direct report exists in the literature), *NAS* Non-traceable Author Statement (i.e., not directly observed for the living, isolated sample, but based on a generally accepted property for the species, or anecdotal evidence). These evidence codes are from the Gene Ontology project [38]

**Table 2:** Project information

MIGS ID	Property	Term
MIGS-31	Finishing quality	Finished
MIGS-28	Libraries Used	3 kb, 8 kb, and fosmid DNA
MIGS-29	Sequencing platforms	ABI3730
MIGS-31.2	Fold coverage	12.5×
	Sequencing quality	Less than one error per 50 kb
MIGS-30	Assemblers	Phrap
MIGS-32	Gene calling method	CRITICA, GLIMMER, GenePRIMP
	Locus tag	Hlac
	GenBank IDs	CP001365, CP001366, CP001367
	GenBank date of release	February 4, 2009
	GOLD ID	Gc00952
	BIOPROJECT	PRJNA18455
	NCBI project ID	18455
	IMG Taxon ID	643692025
MIGS-13	Source material identifier	ATCC 49239, DSM 5036
	Project relevance	Tree of Life, cold adaptation

### Growth conditions and genomic DNA preparation

*H. lacusprofundi*ATCC 49239 was grown in Franzmann medium (180 g NaCl, 75 g MgCl_2_ · 6H2O, 7.4 g MgSO_4_ · 7H_2_O, 7.4 g KCl, 1 g CaCl_2_ · 2H_2_O, 10 g C_4_H_4_O_4_Na_2_ · 6H_2_O per liter, pH 7.4 with addition of 10 ml vitamin solution) [1]. The vitamin solution contained 0.1 g biotin, 0.1 g cyanocobalamin, and 0.1 g thiamine HCl per liter. Cells were grown with shaking at 220 rpm at 4 °C with illumination.

The DNA extraction method was modified from [9]. Cells were grown to OD_600_ = 0.8, collected by centrifugation at 8000 rpm for 10 min at 4 °C, resuspended in 1/20 volume basal salts and lysed by addition of 2 volumes of deionized water and mixing at room temperature. Next, proteinase K was added to a final concentration of 100 μg/ml, mixed gently, and incubated for 1 h at 37 °C. The lysate was extracted using an equal volume of phenol, mixed gently by inverting at room temperature for 5 min, and then spinning at 8000 g for 15 min at 4 °C. The aqueous and interphase was collected and the phenol extraction was repeated twice more. The aqueous and interphase were then dialyzed against TE overnight at 4 °C with one change of buffer. The dialyzed solution was collected and RNase A was added to a final concentration of 50 μg/ml, the solution was mixed and incubated for 2 h at 37 °C with gentle shaking. Proteinase K was added to a final concentration of 100 μg/ml, mixed and incubated for an additional hour at 37 °C. The RNase A and proteinase K steps were repeated. The DNA was then dialyzed overnight against TE at 4 °C with one buffer change.

### Genome sequencing and assembly

The genome of *H. lacusprofundi* was sequenced at the Joint Genome Institute using a combination of 3 kb, 8 kb, and fosmid DNA libraries. All general aspects of library construction and sequencing were performed at the JGI [10]. Draft assemblies were based on 40,800 total reads. All libraries provided 12.5× coverage. The Phred/Phrap/Consed software package was used for sequence assembly and quality assessment [11–13]. After the shotgun stage, reads were assembled with parallel phrap (High Performance Software, LLC). Possible mis-assemblies were corrected with Dupfinisher [14] or transposon bombing of bridging clones (Epicentre Biotechnologies, Madison, WI). Gaps between contigs were closed by editing in Consed, custom primer walk or PCR amplification (Roche Applied Science, Indianapolis, IN). A total of 1722 additional reactions were necessary to close gaps and to raise the quality of the finished sequence. The completed genome sequence of *H. lacusprofundi* contains 54,250 reads, achieving an average of 11.8× and 13.8× coverage in the chromosomes per base with an error rate of less than 1 in 50,000 bp.

### Genome annotation

Protein-coding genes were identified using a combination of CRITICA [15] and Glimmer [16] followed by a round of manual curation using the JGI GenePRIMP pipeline [17]. GenePRIMP points out cases where gene start sites may be incorrect based on alignment with homologous proteins. It also highlights genes that appear to be broken into two or more pieces, due to a premature stop codon or frameshift, and genes that are disrupted by transposable elements. All of these types of broken and interrupted genes are labeled as pseudogenes. Genes that may have been missed by the gene calling programs are also identified in intergenic regions. The predicted CDSs were translated and used to search the National Center for Biotechnology Information nonredundant database, UniProt, TIGRFam, Pfam, PRIAM, KEGG, COG, and Interpro databases. Signal peptides were identified with SignalP [18], and transmembrane helices were determined with TMHMM [19]. CRISPR elements were identified with the CRISPR Recognition Tool [20]. Paralogs are hits of a protein against another protein within the same genome with an e-value of 10^−2^ or lower. The tRNAScanSE tool [21] was used to find tRNA genes. Additional gene prediction analysis and manual functional annotation was performed within the Integrated Microbial Genomes Expert Review (IMG-ER) [22] and HaloWeb [23] platform.

## Genome properties

The genome of *H. lacusprofundi* consists of two chromosomes of length 2,735,295 bp (Chromosome 1) and 525,943 bp (Chromosome 2 or pHL500) and one plasmid of length 431,338 bp (pHL400) (Table 3). The map of the genome is available on HaloWeb [24]. Partial sequence was obtained from a second smaller plasmid, but it appeared to be present in a minority of the cells and its complete sequence could not be determined. The GC content of the large chromosome (67 %) is larger than those of the small chromosome (57 %) and the plasmid (55 %). There are 2801 genes on the large chromosome, 522 genes on the smaller chromosome, and 402 genes on the plasmid. Two of the ribosomal RNA operons are on the large chromosome and one is found on the smaller chromosome. The properties and statistics of the genome are summarized in Table 4, and genes belonging to COG functional categories are listed in Table 5.

**Table 3:** Summary of genome: two chromosomes and one plasmid

Label	Size (Mb)	Topology	INSDC identifier	RefSeq ID
Chromosome 1	2.74	circular	CP001365.1	NC012029.1
Chromosome 2 (pHL500)	0.53	circular	CP001366.1	NC012028.1
Plasmid (pHL400)	0.43	circular	CP001367.1	NC012030.1

**Table 4:** Genome statistics

Attribute	Value	% of Total
Genome size (bp)	3,692,576	100.00 %
DNA coding (bp)	3,199,417	86.64 %
DNA G + C (bp)	2,362,214	63.97 %
DNA scaffolds	3	
Number of replicons	3	
Extrachromosomal elements	1	
Total genes	3725	100.00 %
Protein coding genes	3665	98.39 %
RNA genes	60	1.61 %
Pseudo genes	105	2.82 %
Genes in internal clusters	2009	53.93 %
Genes with function prediction	2143	57.53 %
Genes assigned to COGs	2226	59.76 %
Genes with Pfam domains	2162	58.04 %
Genes with signal peptides	396	10.63 %
Genes with transmembrane helices	779	20.91 %
CRISPR repeats	3

**Table 5:** Numbers of genes associated with the 25 general COG functional categories

Code	Value	% age^a^	Description
J	159	4.34	Translation, ribosomal structure and biogenesis
A	0	0.00	RNA processing and modification
K	136	3.71	Transcription
L	226	6.17	Replication, recombination and repair
B	4	0.11	Chromatin structure and dynamics
D	27	0.74	Cell cycle control, Cell division, chromosome partitioning
V	27	0.74	Defense mechanisms
T	104	2.84	Signal transduction mechanisms
M	68	1.86	Cell wall/membrane biogenesis
N	28	0.76	Cell motility
U	30	0.82	Intracellular trafficking and secretion
O	111	3.03	Posttranslational modification, protein turnover, chaperones
C	156	4.26	Energy production and conversion
G	113	3.08	Carbohydrate transport and metabolism
E	227	6.19	Amino acid transport and metabolism
F	73	1.99	Nucleotide transport and metabolism
H	122	3.33	Coenzyme transport and metabolism
I	62	1.69	Lipid transport and metabolism
P	146	3.98	Inorganic ion transport and metabolism
Q	33	0.90	Secondary metabolites biosynthesis, transport and catabolism
Z	0	0.00	Cytoskeleton
W	0	0.00	Extracellular structures
Y	0	0.00	Nuclear structure
R	362	9.88	General function prediction only
S	214	5.84	Function unknown
-	1439	39.26	Not in COGs

^a^The total is based on the total number of protein coding genes in the annotated genome

## Conclusions

The *Halorubrum lacusprofundi* genome sequence is the first established from a cold-adapted haloarchaeon. The genome has features typical of halophilic Archaea, including high G + C-content, large extrachromosomal replicons, and eukaryotic-like DNA replication and transcription genes. Encoded proteins are highly acidic with properties that suggest looser packing and greater flexibility important for function at cold temperatures [25–28]. *H. lacusprofundi* co-exists in a community of three major haloarchaea in Deep Lake, Antarctica [29, 30].

## Abbreviations

TE: Tris-EDTA buffer
CRITICA: Coding region identification tool invoking comparative analysis
PRIAM: PRofils pour l’Identification Automatique du Métabolisme
KEGG: Kyoto Encyclopedia of Genes and Genomes
COG: Clusters of Orthologous Groups
TMHMM: Transmembrane hidden Markov model
CRISPR: Clustered regularly interspaced short palindromic repeats
